# Official invitation letters to promote male partner attendance and couple voluntary HIV counselling and testing in antenatal care: an implementation study in Mbeya Region, Tanzania

**DOI:** 10.1186/s12978-015-0084-x

**Published:** 2015-10-15

**Authors:** Laura F. Jefferys, Philo Nchimbi, Paulina Mbezi, Julius Sewangi, Stefanie Theuring

**Affiliations:** Institute of Tropical Medicine and International Health, Charité-Universitätsmedizin, Berlin, Germany; PMTCT Program Mbeya Region, Ministry of Health and Social Welfare, Mbeya, Tanzania; Regional AIDS Control Program Mbeya, Ministry of Health and Social Welfare, Mbeya, Tanzania

**Keywords:** Invitation letters, Male involvement, ANC, CVCT, HIV, PMTCT, Tanzania

## Abstract

**Background:**

The benefits of male partner involvement in antenatal care (ANC) and prevention of mother-to-child transmission of HIV (PMTCT) for maternal and infant health outcomes have been well recognised. However, in many sub-Saharan African settings, male involvement in these services remains low. Previous research has suggested written invitation letters as a way to promote male partner involvement.

**Methods:**

In this implementation study conducted at three study sites in southwest Tanzania, acceptability of written invitation letters for male partners was assessed. Pre-study CVCT rates of 2–19 % had been recorded at the study sites. Pregnant women approaching ANC without a male partner were given an official letter, inviting the partner to attend a joint ANC and couple voluntary counselling and testing (CVCT) session. Partner attendance was recorded at subsequent antenatal visits, and the invitation was repeated if the partner did not attend. Analysis of socio-demographic indices associated with male partner attendance at ANC was also performed.

**Results:**

Out of 318 women who received an invitation letter for their partner, 53.5 % returned with their partners for a joint ANC session; of these, 81 % proceeded to CVCT. Self-reported HIV-positive status at baseline was negatively associated with partner return (*p* = 0.033). Male attendance varied significantly between the rural and urban study sites (*p* < 0.001) with rates as high as 76 % at the rural site compared to 31 % at the urban health centre. The majority of women assessed the joint ANC session as a favourable experience, however 7 (75 %) of women in HIV-positive discordant or concordant relationships reported problems during mutual disclosure. Beneficial outcomes reported one month after the session included improved client- provider relationship, improved intra-couple communication and enhanced sexual and reproductive health decision-making.

**Conclusion:**

Official invitation letters are a feasible intervention in a resource limited sub-Saharan African context, they are highly accepted by couple members, and are an effective way to encourage men to attend ANC and CVCT. Pre-intervention CVCT rates were improved in all sites. However, urban settings might require extra emphasis to reach high rates of partner attendance compared to smaller rural health centres.

## Background

Although much progress has been made towards reaching the United Nations Millennium Development Goals 4,5 and 6, many Sub-Saharan African countries still have high HIV incidence, high maternal and infant mortality, high attrition from prevention of mother-to-child transmission of HIV (PMTCT) services and suboptimal use of health facilities for delivery [1–3]. Male involvement in antenatal care (ANC) is seen as an increasingly valuable way to improve a number of these health indicators [4–6].

Encouraging results from male involvement in ANC have been shown in a number of studies. Male partner involvement (MPI) has been associated with more women delivering with a skilled birthing assistant [7, 8], and increased condom use is seen when couple voluntary counselling and HIV testing (CVCT) is included as part of the ANC visit [9, 10]. Furthermore, evidence from qualitative research has shown that the absence of male partner involvement in ANC and PMTCT can create a barrier for women to access these services [11].

Involving men in ANC is the gateway to their involvement in PMTCT [3, 5]. Studies have shown that MPI, in PMTCT, is associated with improved maternal adherence to antiretroviral therapy (ART) [10], infant feeding as per medical advice [12, 13] and retention in PMTCT programmes [2, 14]. Additionally, a study in Kenya showed an association between MPI and reduced HIV incidence in infants born to HIV positive women [15].

In many settings, HIV related health outcomes have been described as poorer for men than for women, with fewer men testing for HIV and more men initiating ART at a more advanced disease stage [16, 17]. Creating a channel for men to gain improved access to health education, VCT, and links to ART could impact on their health outcomes. A shift in perspective of PMTCT from the mother and infant to the family as a complete unit has been shown to improve ART adherence [18], and is recommended as a way for policy makers to encourage the inclusion of more men in HIV programmes [5].

Qualitative research has revealed that although men are generally interested in participating in ANC and PMTCT, in practice, numerous barriers prevent their involvement. Social norms can prevent women from asking their partners to attend ANC or for men to concede to attending [19]. ANC has been considered an arena for women, with predominately female staff, and reports of hostile reactions towards male partners if they attend [20]. Clinic opening hours and long waiting time conflict with male partner work commitments and, for some, the pressure to earn an income is greater than that of attending ANC [20, 21]. Formative research from different sub-Saharan African countries has recommended interventions to increase male partner attendance or involvement, such as opening clinics at evenings or weekends, peer-to-peer ANC/PMTCT education led by men, or home based CVCT during pregnancy [14, 22, 23]. Official written letters of invitation requesting male partner attendance ANC has been reported by both men and women as a good way to encourage male participation. Invitations are easy and cheap to implement and can overcome social normative barriers by removing the need for women to directly ask their partners to attend ANC, and the official nature of the invite invokes a certain authority which is reported to be respected by male partners [20, 21-24, 25]. However, further research is still needed to evaluate the effectiveness of written invitations in different settings [26].

In Tanzania, the national HIV prevalence is 5.3 %, while in our study area of Mbeya Region, it is higher at 9 % [27]. An estimated 43,000 new paediatric HIV infections annually contribute to almost a fifth of all new infections in the country [27]. Seventy per cent of Tanzanian women who tested HIV-positive during pregnancy received some form of ART regimen during their pregnancy and 50 % of HIV-exposed infants received ART prophylaxis. Although this is higher than the average for women receiving ART during pregnancy in the African priority countries (65 %), it represents a 20 % gap from the global 2015 target of 90 % [28, 29]. Furthermore, this perspective on the data does not take into account the estimated 65 % of women in lower and middle-income countries who are not tested for HIV during their pregnancy at all [28]. The lack of male participation in ANC and PMTCT has been identified in the National Strategic Plan as a key barrier to achieving PMTCT in Tanzania [27]. In Tanzania 21 % of male partners reportedly tested for HIV during their partner’s pregnancy in 2010, and strategies are being implemented to promote CVCT alongside family planning for both men and women, with the aim to increase male partner testing during pregnancy to 50 % by 2015 [27, 29]. Given the low rates of MPI and CVCT combined with the increasing need to find effective strategies to improve them, we designed an implementation study to assess the acceptability and effectiveness of written invitations for male partners to attend joint ANC and CVCT in Tanzania. Secondary objectives of the study include analysis of socio-demographic indices associated with male partner attendance and evaluation of the repercussions of MPI for the women.

## Methods

This implementation study was conducted in Mbeya Region, southwest Tanzania. Data was collected from a prospective, longitudinal cohort at three health centres at different locations in Mbeya Region. The centres were selected purposively and based on a maximum variation sampling approach to evaluate male involvement in urban, rural and border town settings, in order to encompass a wide range of socio-demographic indices according to these different settings (respectively: Ruanda health centre, Mbeya City, Makongolosi dispensary, Chunya District, Tunduma health centre, Mbozi District). All three centres are free of charge, primary health care facilities, offering HIV treatment for the general population, pregnant and breastfeeding women. Early infant diagnosis by PCR is done at the tertiary referral hospital in Mbeya. Study enrolment was conducted between March 2013 and June 2013, during this time 1492 women presented for ANC at Ruanda, 697 at Tunduma and 122 at Makongolosi. Prior to the study period few male partners joined their partner for CVCT. Available data from the clinic registers reported that over the preceding three-month period 1.7 % of partners of all new ANC clients at Ruanda health centre had received CVCT, and 2.2 % at Tunduma health centre. Data from Makongolosi was accessible for male attendance from 2012 and showed a rate of 18.5 %.

Women attending ANC for the first time during their current pregnancy were recruited into the study, after written informed consent had been obtained. Eligibility criteria included a confirmed pregnancy and general accessibility of the partner, which was assessed by asking the women if their partner was permanently living away from the area or in a health condition that would not allow him accompany her to ANC services. Women were excluded if their partner attended the first ANC visit with them. Three separate questionnaires were developed for the interviews; these had been pre-tested on clinic attendees and adjusted accordingly by the investigators. Research assistants were recruited and received training from the principle investigator. In order to prevent the study procedures from interfering with running of the clinic, the research assistants were not routine clinic staff. Interviews were conducted after routine ANC sessions in a separate room and not in the presence of the ANC nursing staff or the male partner. During the routine ANC sessions, women were offered opt-out HIV testing as a standard ANC procedure.

After attending the first ANC visit, a baseline questionnaire was filled out by a research assistant to gather socio-demographic information about the study participant and their partner, including information about intimate partner violence (IPV) and knowledge of HIV status. Formal employment was defined as receiving a salary from another body or company. IPV included any physical, emotional or financial abuse reported by the woman. Results of HIV tests performed within the study sites were not known to the research team, therefore data on participant and partner HIV status’ were self-reported by participants.

At the end of the baseline interview, women were given a written invitation letter for their male partner requesting their presence at the next routine ANC visit. This letter explained that information on pregnancy and parenthood and other important health issues would be given. It did not state that an HIV test would be offered. The letter was signed by the regional medical officer and was courteous and formal.

If the partner attended the next visit as requested, a joint antenatal session would take place during which CVCT was provided, if agreed to, by the couple. After this joint session the research assistant would interview the woman, collecting information about the partner’s reaction on receiving an invitation letter and information about the session itself. If either of the couple did not want to take part in CVCT, VCT would be offered individually as part of standard procedures. CVCT included intensive pre- and post- test counselling of both partners and support in case of any problems during mutual status disclosure.

If the partner had not accompanied the woman to this second ANC visit, information was obtained regarding the reasons for his non-attendance and a further letter of invitation was given with a new appointment for a partner session.

After the third ANC visit recruited women were again interviewed by the research assistant. Those who had already been accompanied by their partner at the second visit gave information pertaining to any positive or negative repercussions and longer-term outcomes of the joint session. Women whose partners had not attended the second visit were asked if they attended at this third visit, and were then interviewed either regarding their opinion on the joint ANC and CVCT session, or regarding reasons for the partner to reject participation. No additional invitation letter was handed out for non-attending partners after the third visit.

During the study period intermittent community sensitisation on HIV and PMTCT occurred as part of routine government public health initiatives, which included community leader involvement and radio broadcasts encouraging VCT. This was the case for all the three study site areas. HIV-positive ANC clients lost to follow-up were routinely traced in community outreach programs in our study setting.

### Statistical methods

Data analysis was performed using STATA version 13.1. To compare the socio-demographic indices across three different health centres, Kruskal-Wallis one-way analysis of variance was used for non-parametric continuous outcomes, and Fisher’s exact test was used for categorical outcomes due to low cell values in the contingency table (<5 in one cell). To compare data between the women and their partners, two-sample Wilcoxon rank-sum test was used for medians, as the data was non-parametric, and two-sample test of proportion was used for proportions.

For the bivariate analysis of socio-demographic indices on partner attendance variables, chi-square or Fishers exact test was used depending on the cell value.

A multiple logistic regression model was used for the multivariate analysis of factors associated with partner attendance and included the variables self-reported baseline HIV status, age, marital status, health facility, media exposure, IPV, partner employment and travel time to clinic. These variables were of interest as they were either significant or close to significant (*p* < 0.06) in the bivariate analysis. Questionnaire responses from Makongolosi for the variable ‘previous partner involvement in ANC’ was 100 % uniform and considered to have been collected incorrectly and was therefore not included in the regression model.

A significance level of <0.05 and a 95 % confidence interval (CI) was used throughout.

In addition to quantitative data, qualitative free-text was recorded to allow participants to expand on events that may have occurred after the joint session. These open-ended responses were categorised into relevant groups to allow for quantitative assessment.

### Ethical considerations

The Mbeya Medical Research and Ethics Committee in Tanzania provided ethical approval for the time period of recruitment, data collection, and follow up. All participants gave informed written consent. The research assistant’s training included participant confidentiality, and all data was recorded anonymously. Potential adverse outcomes connected to male involvement CVCT, like increased IPV, were closely screened during our study in order to respond appropriately if needed, and ANC staff was routinely trained in mediation and conflict management during CVCT in these healthcare settings.

## Results

Between March and June 2013, 318 women were recruited into the study: 97 at Ruanda health centre, 101 at Tunduma health centre and 120 at Makongolosi dispensary. In total 237 (74.5 %) of the women had at least two visits and 143 (45 %) had three. The total male return rate during the study period was 170 (53.5 %).

### Baseline socio-demographic indices of all study participants

The women were aged between 14 and 44 years (median 23) and presented at a median gestational week of 22. Many of the socio-demographic indices varied significantly between the health centres (Table 1). Women were oldest in Ruanda with a median age of 24 years at the urban setting Ruanda, and youngest at 22 years in the rural setting of Makongolosi (*p* = 0.004). Median travel time to clinic was highest at 30 min for Ruanda, lowest at ten minutes for Tunduma (*p* < 0.001).

**Table 1:** Comparison of study participant characteristics by health centre

Characteristics of study participants	Sub-categories	Total	Makongolosi	Tunduma	Ruanda	*P* value
Total n		318	120	101	97	-
Age (years) median (Range)		23 (14–44)	22 (14–38)	23 (16–42)	24 (18–44)	0.004^c^
Gestation (wks) median (Range)		22 (4–36)	20 (4–36)	28 (16–36)	20 (7–34)	<0.001^c^
Travel time to clinic (minutes) median (range)		15 (1–360)	13.5 (1–120)	10 (5–20)	30 (5–360)	<0.001^c^
Travel cost (Shillings) median (range)		800 (0–4000)	^a^	300 (0–1000)	800 (0–4000)	<0.001^c^
Literate n (%)		270 (86)	89 (76)	91 (90)	90 (93)	0.001^d^
Marital status n (%)	Married	236 (74.2)	113 (94.2)	95 (95)	28 (28.9)	<0.001^d^
	Partnership	82 (25.8)	7 (5.8)	6 (5.9)	69 (71.1)	-
Religion n (%)	Christian	284 (90.5)	101 (86.3)	92 (91.1)	91 (94.8)	0.116^d^
	Muslim & Other	30 (9.6)	16 (13.7)	9 (8.9)	5 (5.2)	-
Education n (%)	None	49 (15.4)	30 (25)	10 (9.9)	9 (9.3)	0.001^d^
	Primary	203 (63.8)	74 (61.7)	71 (70.3)	58 (59.8)	-
	Secondary	66 (20.8)	16 (13.3)	20 (19.8)	30 (30.9)	-
Employment n (%)	None	59 (18.9)	10 (8.5)	27 (27.6)	22 (22.7)	<0.001^d^
	Formal	8 (2.6)	-	1 (1.0)	7 (7.2)	-
	Self-employed	245 (78.3)	107 (91.5)	70 (71.4)	68 (70.1)	-
Children at home N (%)	None	131 (46.2)	32 (37.2)	43 (43.4)	56 (58.3)	0.065^d^
	1_2	110 (39.2)	39 (45.4)	41 (41.4)	31 (31.3)	-
	>2	40 (14.2)	15 (17.4)	15 (15.2)	10 (10.4)	-
Media exposure at home n (%)	None	6 (1.9)	1 (0.8)	1 (1)	4 (4.1)	<0.001^d^
	Radio	190 (59.8)	95 (79.1)	51 (50.5)	44 (45.4)	-
	Media plus^b^	122 (38.4)	24 (20)	49 (48.5)	49 (50.5)	-

^a^Data not available

^b^Media plus: radio + TV +/− Newspaper, or TV or Newspaper only, or TV + Newspaper without radio

*P* values calculated using: ^c^Kruskal-Wallis one-way analysis of variance. ^d^Fishers Exact Test

The majority of the women were literate (86 %), achieved at least primary education (85 %) and were of Christian religion (91 %). Forty six per cent of the women reported having no children at home and 74 % were married. Around 25 % reported some form of IPV, of this abuse 80 % was emotional, 11 % physical and 8 % financial.

Twenty nine per cent of women reported that they did not know their HIV status, and 72 % said that they did not know their partners´ HIV status. Knowledge of partner HIV status varied between the health centres; knowledge of a partner’s HIV status was highest in Tunduma (51 %) and lowest in Makongolosi (10 %) (*p* < 0.001).

The partners of the study participants were aged between 19 and 55 years (median 28), with a median age difference within the couple of four years and relationship duration of three years (Table 2). Almost all the partners had received at least primary education (97 %), were literate (96 %) and self-employed (89 %). The men were, on average, 5 years older than the women, and had a higher literacy rate: 96 % of the men compared to 86 % of the women (both *p* < 0.001), 30 % of the men had received secondary education, as had 21 % of the women (*p* = 0.007). The partners with a secondary education were more likely to be in formal employment (*p* <0.001). Between the study sites partner age, literacy, education and employment differed significantly.

**Table 2:** Comparison of partner characteristics of study participants by health centre

Partner/Relationship characteristics	Sub-categories	Total	Makongolosi	Tunduma	Ruanda	*P* value
Partner’s age (years) median (range)		28 (19–55)	26 (19–55)	28 (19–50)	29 (20–49)	0.020^a^
Age difference (years) median (range)		4 (0–21)	5 (1–21)	4 (1–21)	4.5(0–20)	0.254^a^
Duration of relationship (years) median (range)		3 (0–23)	2 (0.25–23)	3 (0.25–23)	4 (0.25–17)	0.103^a^
Partner literate n (%)		301 (95.9)	109 (91.6)	96 (98)	96 (99)	0.012^b^
Partner’s education n (%)	None	11 (3.5)	9 (7.6)	1 (1.0)	1 (1)	<0.001^b^
	Primary	208 (66.5)	89 (74.8)	69 (70.4)	50 (52.1)	-
	Secondary	94 (30)	21 (17.6)	28 (28.6)	45 (46.9)	-
Partner’s employment n (%)	None	10 (3.2)	1 (0.8)	1 (1.0)	8 (8.3)	<0.001^b^
	Formal	24 (7.7)	1 (0.8)	4 (4.1)	19 (29.8)	-
	Self	278 (89.1)	117 (98.4)	92 (94.9)	69 (71.9)	-

*P* values calculated using: ^a^Kruskal-Wallis one-way analysis of variance. ^b^Fishers Exact Test

### Bivariate analysis of socio-demographic indices associated with partner attendance

Bivariate analysis (Table 3) was performed to assess the association of socio-demographic indices with partner attendance, Partner attendance was associated with a number of characteristics: women under 26 years of age and married women had significantly higher odds ratios (OR) of partner attendance (respectively: OR 1.72 *p* = 0.022 & OR 3.13 *p* < 0.001). Travel time to clinic was associated with borderline significance (OR 1.56 *p* = 0.051).

**Table 3:** Bivariate analysis of factors effecting partner attendance at ANC

Variable	Categories	Study participants N/%	Partner attended N/%	OR	95 % CI	*P* value
Health facility	Makongolosi	120/37.7	91/75.8	7.01	3.57–13.74	<0.001^c^
	Tunduma	101/31.8	49/48.5	2.1	1.16–3.8	0.012^c^
	Ruanda	97/30.5	30/30.9	1	-	-
Age	<26 years	218/68.6	126/57.8	1.74	1.08–2.8	0.022^c^
	≥26 years	100/31.5	44/44	1	-	-
Travel time to clinic	≤15 min	186/58.5	108/58.1	1.56	0.99–2.46	0.051^c^
	>15 min	132/41.5	62/47	1	-	-
Literate	Literate	301/95.9	157/52.2	0.33	0.088–1.22	0.08^c^
	Illiterate	13/4.1	10/76.9	1	-	-
Marital status	Married	236/74.2	143/60.1	3.13	1.81–5.41	<0.001^c^
	Partnership	82/25.8	27/32.9	1	-	-
Religion	Christian	284/90.5	149/52.5	1	-	-
	Muslim + Others	30/9.5	19/63.3	1.07	0.5–2.32	0.86^d^
Partners age	≤26 years	117/38	69/59	1.45	0.91–2.32	0.115^c^
	>26 years	191/62	95/49.7	1	-	-
Partner employment	None	10/3.6	5/50	0.81	0.23–2.85	0.041^e^
	Formal	24/7.7	7/29.2	0.33	0.13–0.834	-
	Self-employed	278/89.1	154/55.4	1	-	-
Number of children at home	None	131/46.6	67/51.2	1	-	-
	1–2	110/39.2	50/45.6	0.79	0.48–1.32	-
	>2	40/14.2	22/55	1.17	0.57–2.38	0.51^e^
IPV	IPV reported	75/24.4	28/37.3	1	-	-
	Not reported	232 75.6	133/57.3	2.26	1.31–3.39	0.003^d^
Media	None	6/1.9	2/33.3	1		0.042^e^
	Radio	190/59.8	112/59	2.87	0.51–16.25	-
	Media plus^a^	122/38.4	56/45.9	1.7	0.297–9.7	-
Previous partner attendance at ANC^b^	Yes	27/13.9	19.4/70.4	4.17	1.63–10.67	0.002^d^
Self-reported HIV status (baseline)	Positive	8/2.6	1/12.5	0.11	0.01–0.92	0.025^e^
	Negative	212/68.6	121/57.1	1	-	-
	Unknown	89/28.8	43/48.3	0.7	0.43–1.16	-

^a^Media plus = radio + TV +/− Newspaper, or TV or Newspaper only, or TV + Newspaper without radio

^b^Data from Tunduma & Ruanda health centres

*P* value calculated using : ^c^Chi-squared test. ^d^ Fishers exact test. ^e^Fishers exact test (homogeneity/equal odds)

Formal sector employment of the partner was associated significantly with non-attendance (OR 0.33 *p* = 0.041), and previous partner attendance at ANC was associated with their re-attendance (OR 4.17 *p* = 0.002).

The different health facilities had a profound effect on partner attendance, the odds of partner attendance at Makongolosi being 7 times higher than at Ruanda (*p* < 0.001).

Having a radio at home, rather than any other combination of media exposure, and the absence of previous IPV were both significantly associated with partner attendance (respectively OR 2.87 *p* = 0.042 and OR 2.26 *p* = 0.003).

### Analysis of the association between HIV status and partner attendance

At baseline, 8 women (2.6 %) reported that they were HIV positive and 5 (1.6 %) reported that their partners were HIV positive. A self-reported positive HIV status by the woman at baseline decreased chances for attendance of the woman at subsequent ANC visits (OR= 0.28, *p* = 0.061), and significantly decreased chances for partner attendance (OR= 0.11, *p* = 0.025). 

### Multivariate analysis of factors associated with partner attendance

Multivariate analysis revealed that partner attendance remained independently associated with being enrolled at the rural health centre Makongolosi (adjusted odds ratio [AOR] 7.5, *p* < 0.001). Self-reported positive HIV status at baseline also remained associated with partner non-attendance (AOR 11.12, *p* = 0.033). All other variables lost significance at this stage of the analysis (Table 4).

**Table 4:** Multivariate analysis of factors effecting partner attendance at ANC

Variable	Categories	AOR for partner attendance	95 % CI	*P* value*
Health facility	Makongolosi	7.55	2.8–20.4	<0.001
	Tunduma	2.67	0.3–39.6	0.06
	Ruanda	1	-	-
Age	<26 years	1.5	0.86–2.6	0.15
	≥26 years	1	-	-
Travel time to clinic	≤15 min	1	-	-
	>15 min	1.004	0.99–1.01	0.406
Marital status	Married	1.09	0.47–2.55	0.84
	Partnership	1	-	-
Partner employment	None	1.8	0.45–7.4	0.396
	Formal	0.83	0.28–2.5	0.74
	Self-employment	1	-	-
Media	None	1	-	-
	Radio	3.72	0.33–42.5	0.289
	Media plus^a^	3.44	0.3–39.6	0.322
IPV	IPV reported	1.22	0.63–2.35	0.552
	Not reported	1	-	-
Self-reported HIV status (baseline)	Positive	0.09	0.01–0.82	0.033
	Negative	1	-	-
	Unkown	1.17	0.57–2.38	0.67

^a^Media plus = radio + TV +/− Newspaper, or TV or Newspaper only, or TV + Newspaper without radio

*P values calculated using a multiple logistic regression model

### Acceptability and effectiveness of invitation letters

The letters were well accepted, 98 % of women who returned to clinic reported they had handed the letter to their partners. Male partners were supportive after having received a written invitation.

Across the three study sites the partner attendance rate was 53.5 %. Women attending ANC in Makongolosi showed the highest response, with 75.8 % returning with partners, while in Tunduma, the partner return rate was 48.5 %. Partner attendance was lowest in the urban setting of Ruanda health centre 31 % (Table 3).

When the partner attended a joint ANC session, 81 % of the couples received CVCT, while in the remaining 19 % only the women tested. Immediately after the session 95 % of women reported that the counsellor was helpful, 91 % stated the experience was good and (90 %) stated that there were no difficulties during mutual disclosure of HIV status.

Of women who had had a partner in attendance at the second visit, 115 attended a follow-up session roughly one month later. Seventy-five (71 %) reported positive events resulting from the joint ANC session whilst only nine (8 %) reported negative events. Positive events related to an improved relationship between the partner or the couple and the health services (40 %), improved communication and support between the couple (28 %) and an exposure to health education for the couple (23 %). Around 95 % of women stated that the joint session helped to improve their role in decision-making regarding ANC, family planning and sexual and reproductive health.

Of the negative events reported, five (56 %) occurred after one or both of the couple tested positive for HIV. These negative events included separation, blame and problems with negotiating safe sex. The majority of IPV was also reported after a couple had received a positive HIV result (4; 67 %), two reported emotional abuse and two reported financial abuse. Three of these couples were discordant, with a positive male partner in two cases, and one was a concordant couple.

The expected future events cited by the women were reduced anxiety about HIV testing and disclosure, improved communication within the couple, and improved relationship between the partner and the service providers.

Eighty-eight per cent of women thought they would have future joint ANC sessions and 95 % would recommend others to participate in joint ANC sessions. Fifty-five per cent of women shared with either family or friends that they had attended a joint session.

Of the 170 male partners attending ANC, 79 % of them did so after the first invite and 21 % attended after the second invitation. The most commonly stated reason for male non-attendance was visiting un-well relatives or attending a funeral (42 %), followed by work commitments (33.3 %). Those who stated work commitments as a reason for non-attendance had higher of odds of being in formal employment compared to self-employment (OR 3.13 *p* = 0.097). Marital conflict was also given as a reason for non-attendance (14 %).

### Self-reported HIV results post-CVCT

After having attended CVCT during a joint ANC session, six women reported HIV positive results and eight stated that their partner tested positive; this corresponds to a prevalence of 4.3 % for the women and 7.8 % for the men for the study participants who underwent CVCT. Five couples were reported as concordant and three couples discordant, with two of these having a positive male partner. Seven (75 %) of the women in a HIV positive concordant or discordant relationship stated that there was a difficulty such as disappointment with results or conflict during mutual disclosure, despite this 38 % of them stated that the joint ANC session had been overall a good experience.

## Discussion

Our study assessed male partner attendance at joint ANC sessions after providing women attending ANC a written invitation for their partners. Half of the women in our study returned to ANC with their male partner, and of these 81 % received CVCT. Data available from the pre-study period showed between 2 and 19 % of partners received CVCT at ANC. Although we cannot make a direct comparison between this historical data and the existing study results, the vastly higher numbers of male partners attending ANC after invitation is encouraging.

Our results also show higher partner attendance at ANC than some of the previous studies assessing written invitations. For example reported male attendance at ANC following invitation letters was 16 % in Uganda [30], 26–35 % in South Africa [26], 33 % in Tanzania [31] and 36 % in Kenya [22]. In studies where women were encouraged to verbally invite their partner, male attendance at ANC was lower: 11 % in Kenya [12] and 12 % in Tanzania [10]. Outside of the context of ANC, the effectiveness of invitation letters for CVCT has also been assessed: results from a multi-centre study in Zambia and Ruanda showed 14 % of couples attended for CVCT after written invitation was given to either the couple together or to an individual couple member [23].

Our study showed high levels of acceptability of the invitations by the women, with almost 100 % giving the letter to their partner. Although reporting bias must be taken into account, this is an important finding, because high acceptance of the intervention among women is a precondition for its success. The majority of women reported that joint ANC sessions were a good experience and that positive events occurred afterwards. Discussing difficult topics together with a counsellor during the joint ANC session can be a useful platform to aid future discussions in the home and almost all the women in our study reported they felt that their role in sexual and reproductive decision-making had improved after the session. Over half had told family or friends that they had attended a joint ANC session, this should facilitate the normalisation of partner attendance at ANC.

Three quarters of the women who reported an HIV positive result after CVCT reported problems during disclosure, with some reports of negative events after the session such as IPV and separation. This highlights the on-going difficulties with disclosure of a positive HIV result despite receiving counselling from trained health professionals. The fears that have been described in qualitative research regarding positive HIV result disclosure to partners, especially during a vulnerable time such as pregnancy, are not necessarily removed by CVCT, and therefore more time and support for women, or their partners, who test positive should be available when offering CVCT services [32].

Our written invitations did not include information about receiving an HIV test, as this has been reported as a deterrent to attending joint ANC sessions [24, 31]. Contradicting this, other studies have shown that receiving an HIV test can encourage men to attend ANC, and a study from South Africa that included information about CVCT in their invitation letter showed improved partner attendance rates when compared to a letter without information about CVCT [26, 33]. Increased levels of community knowledge through sensitisation strategies and acceptance of routine CVCT at ANC should decrease any fear of HIV testing that could maintain barriers against men attending ANC. This is necessary to avoid men feeling coerced into testing during ANC, which has been reported during ANC in Zambia [34].

We found significant variation in partner attendance across our study sites: the lowest (31 %) in the urban health clinic and the highest (76 %) the rural clinic. This difference remained independently significant in the multiple regression model. The socio-demographic indices of the women varied significantly between the health centres: women in the urban clinic were older, had a longer travel time to clinic, higher level of secondary education and were more likely to have partners in formal employment. The rural clinic providing health care to a smaller community had the highest partner attendance in our study. It is likely in the smaller community that the participants, and perhaps also their partners, knew the ANC staff. This could have contributed to the higher rates of partner attendance seen in this rural setting as it has been reported that written invitations are more effective if given by someone that the couple knew personally, rather than a stranger [23]. Although few studies in this subject area stratify results by residence, one cross-sectional study retrospectively evaluating male participation in PMTCT found no difference in male attendance between men residing in rural or urban areas [25]. Still, many of the invitation studies have been based in urban areas, with results that correlate with the lower rates from our urban setting [22, 26, 30, 31]. Given that over half the women in sub-Saharan Africa live in rural areas, care may need to be taken when generalising the level of effectiveness of invitations from urban studies to rural populations [35].

Analysis of socio-demographic indices showed that many variables were associated with partner attendance and include the following: younger women, those who were married, not reporting IPV, having a partner who previously attended ANC and a partner in non-formal employment. These variables lost significance in the multivariate analysis. However being enrolled in the rural health centre remained significantly associated with male attendance in the multivariate analysis, and baseline self-reported positive HIV status remained associated with male non-attendance.

High attrition rates after a diagnosis of HIV and before receiving HIV care has been clearly acknowledged in other studies [36, 37]. By documenting self-reported HIV status in this study, we could observe the association between self-reported HIV positivity at baseline and non-return to ANC, either alone or with a partner. These women, unless they presented to alternative ANC services, represent missed opportunities for PMTCT and HIV care. Fear of disclosure during CVCT could have acted as a barrier for these women to return to ANC.

Self-reported HIV positive status was lower than regional HIV prevalence data, this may have been due to under-reporting by study participants and could reflect participants’ fear of disclosure and concerns over confidentiality. At baseline, 29 % of women stated they did not know their own HIV status and 72 % did not know their partners status. This knowledge gap represents a potential risk for both horizontal and vertical HIV infection. Therefore CVCT during ANC is clearly a valuable intervention for our study population, confirming similar insights from other settings: a South African study from 2014 showed that 40 % of recently pregnant women did not know their partners’ HIV status, even after the pregnancy [38].

We suggest that there is value in repeating the invitation to partners, as 21 % of ANC-attending male partners had come only after a second invitation. Work commitments were cited by one third of the women as the reason why her partner did not attend ANC, and 42 % of partners were visiting un-well relatives or attending a funeral. Irrespective to barriers of male attendance being related to their physical absence from the home or to social normative behaviour, there seems to be a benefit in further motivation by health staff, thus providing partners with multiple opportunities to attend.

### Strengths and limitations

By evaluating the acceptability of official invitation letters in three different healthcare scenarios, our study provides a long-needed addition to the current understanding of methods to improve male participation in ANC, and for their systematic implementation. Yet, some limitations were faced during this research.

We were unable to follow up the women who did not continue in the study, while information as to whether they continued ANC at another facility, or not at all, would be valuable to understand the magnitude of loss to follow up. However, the initially recruited overall cohort was sufficiently large to encounter loss-to-follow up with respect to valid study outcomes.

HIV results were self-reported by study participants, and under- or over-reporting may have occurred. This limits the conclusions that can be made regarding associations with HIV status and male partner attendance, and while this was not one of our outcome parameters, we accept that the prevalence of HIV in the cohort is unlikely to be accurate.

Uniformity of data collected for one variable at Makongolosi study site meant that we could not include this variable in the multivariate analysis. This site would have benefitted from more supervision throughout the study period to prevent any data errors occurring.

As this study was not conducted as a randomised controlled trial, we cannot draw conclusions about effectiveness. Nonetheless, given the large effect of the intervention compared to historical data, our findings are still assumed to be highly important for developing strategies to increase partner involvement.

## Conclusions

This study demonstrated that written invitations for male partners to attend joint ANC and CVCT were well accepted by women attending ANC in Mbeya Region, Tanzania, and resulted in more than half of the women returning with their partner at a subsequent ANC visit.

We found significant differences in male attendance between our urban and rural settings, with potential implications for interventions aimed at increasing male attendance at ANC in different settings. Additional approaches maybe required to achieve higher partner attendance in areas with lower response rates to invitations, such as flexible opening hours or targeted community sensitization. The strongly improved partner return rates in all sites when compared to pre-study partner return rates imply that invitation letters are a valuable measure that can be implemented in a sub-Saharan African ANC/PMTCT setting to improve male participation in these services.

## References

[1] Chilongozi D, Wang L, Brown L, Taha T, Valentine M, Emel L (2008). Morbidity and mortality among a cohort of human immunodeficiency virus type 1-infected and uninfected pregnant women and their infants from Malawi, Zambia, and Tanzania. Pediatr Infect Dis J.

[2] Chinkonde JR, Sundby J, Martinson F (2009). The prevention of mother-to-child HIV transmission programme in Lilongwe, Malawi: why do so many women drop out. Reprod Health Matters.

[3] World Health Organisation (WHO) (2010). PMTCT strategic vision 2010–2015: preventing mother-to-child transmission of HIV to reach the UNGASS and Millennium Development Goals.

[4] Jennings L, Na M, Cherewick M, Hindin M, Mullany B, Ahmed S (2014). Women’s empowerment and male involvement in antenatal care: analyses of Demographic and Health Surveys (DHS) in selected African countries. BMC Pregnancy Childbirth.

[5] World Health Organisation (WHO) (2012). Male involvement in the prevention of mother-to-child transmission of HIV.

[6] Betancourt TS, Abrams EJ, McBain R, Fawzi MC (2010). Family-centred approaches to the prevention of mother to child transmission of HIV. J Int AIDS Soc.

[7] Parkhurst JO, Rahman SA, Ssengooba F (2006). Overcoming access barriers for facility-based delivery in low-income settings: insights from Bangladesh and Uganda. J Health Popul Nutr.

[8] Ediau M, Wanyenze RK, Machingaidze S, Otim G, Olwedo A, Iriso R (2013). Trends in antenatal care attendance and health facility delivery following community and health facility systems strengthening interventions in Northern Uganda. BMC Pregnancy Childbirth.

[9] Allen S, Meinzen-Derr J, Kautzman M, Zulu I, Trask S, Fideli U (2003). Sexual behavior of HIV discordant couples after HIV counseling and testing. AIDS.

[10] Msuya SE, Mbizvo EM, Hussain A, Uriyo J, Sam NE, Stray-Pedersen B (2008). Low male partner participation in antenatal HIV counselling and testing in northern Tanzania: implications for preventive programs. AIDS Care.

[11] Gourlay A, Birdthistle I, Mburu G, Iorpenda K, Wringe A (2013). Barriers and facilitating factors to the uptake of antiretroviral drugs for prevention of mother-to-child transmission of HIV in sub-Saharan Africa: a systematic review. J Int AIDS Soc.

[12] Farquhar C, Kiarie JN, Richardson BA, Kabura MN, John FN, Nduati RW (2004). Antenatal couple counseling increases uptake of interventions to prevent HIV-1 transmission. J Acquir Immune Defic Syndr.

[13] Engebretsen IM, Moland KM, Nankunda J, Karamagi CA, Tylleskär T, Tumwine JK (2010). Gendered perceptions on infant feeding in Eastern Uganda: continued need for exclusive breastfeeding support. Int Breastfeed J.

[14] Nyondo AL, Chimwaza AF, Muula AS (2014). Stakeholders’ perceptions on factors influencing male involvement in prevention of mother to child transmission of HIV services in Blantyre, Malawi. BMC Public Health.

[15] Aluisio A, Richardson BA, Bosire R, John-Stewart G, Mbori-Ngacha D, Farquhar C (2011). Male antenatal attendance and HIV testing are associated with decreased infant HIV infection and increased HIV-free survival. J Acquir Immune Defic Syndr.

[16] Mills EJ, Beyrer C, Birungi J, Dybul MR (2012). Engaging men in prevention and care for HIV/AIDS in Africa. PLoS Med.

[17] (CDC) CfDCaP (2013). Differences between HIV-Infected men and women in antiretroviral therapy outcomes - six African countries, 2004–2012. MMWR Morb Mortal Wkly Rep.

[18] Byakika-Tusiime J, Crane J, Oyugi JH, Ragland K, Kawuma A, Musoke P (2009). Longitudinal antiretroviral adherence in HIV+ Ugandan parents and their children initiating HAART in the MTCT-Plus family treatment model: role of depression in declining adherence over time. AIDS Behav.

[19] Falnes EF, Moland KM, Tylleskär T, de Paoli MM, Msuya SE, Engebretsen IM (2011). “It is her responsibility”: partner involvement in prevention of mother to child transmission of HIV programmes, northern Tanzania. J Int AIDS Soc.

[20] Mohlala BK, Gregson S, Boily MC (2012). Barriers to involvement of men in ANC and VCT in Khayelitsha, South Africa. AIDS Care.

[21] Theuring S, Mbezi P, Luvanda H, Jordan-Harder B, Kunz A, Harms G (2009). Male involvement in PMTCT services in Mbeya Region, Tanzania. AIDS Behav.

[22] Osoti AO, John-Stewart G, Kiarie J, Richardson B, Kinuthia J, Krakowiak D (2014). Home visits during pregnancy enhance male partner HIV counselling and testing in Kenya: a randomized clinical trial. AIDS.

[23] Allen S, Karita E, Chomba E, Roth DL, Telfair J, Zulu I (2007). Promotion of couples’ voluntary counselling and testing for HIV through influential networks in two African capital cities. BMC Public Health.

[24] Nyondo AL, Muula AS, Chimwaza AF (2013). Assessment of strategies for male involvement in the prevention of mother-to-child transmission of HIV services in Blantyre, Malawi. Glob Health Action.

[25] Byamugisha R, Tumwine JK, Semiyaga N, Tylleskär T (2010). Determinants of male involvement in the prevention of mother-to-child transmission of HIV programme in Eastern Uganda: a cross-sectional survey. Reprod Health.

[26] Mohlala BK, Boily MC, Gregson S (2011). The forgotten half of the equation: randomized controlled trial of a male invitation to attend couple voluntary counselling and testing. AIDS.

[27] Tanzania Commission for AIDS. Tanzania Third National Multi-Sectoral Strategic Framework for HIV and AIDS (2013/4-2017/8) 2013. http://www.tacaids.go.tz/index.php?option=com_content&view=article&id=44:nmsf&catid=25:strategic-documents&Itemid=140. Accessed Jan 5 2015

[28] World Health Organisation (WHO) (2013). Global update on HIV treatment 2013: results, impact and opportunities, June 2013 Brief summary.

[29] The United Republic of Tanzania Ministry of Health and Social Welfare. Tanzania Elimination of Mother To Child Transmission of HIV Plan, 2012–2015 http://www.emtct-iatt.org/wp-content/uploads/2012/11/Costed-eMTCT-Plan-Final-Nov-20121.pdf. Accessed 5 Jan 2015

[30] Byamugisha R, Åstrøm AN, Ndeezi G, Karamagi CA, Tylleskär T, Tumwine JK (2011). Male partner antenatal attendance and HIV testing in eastern Uganda: a randomized facility-based intervention trial. J Int AIDS Soc.

[31] Becker S, Mlay R, Schwandt HM, Lyamuya E (2010). Comparing couples’ and individual voluntary counseling and testing for HIV at antenatal clinics in Tanzania: a randomized trial. AIDS Behav.

[32] Rujumba J, Neema S, Byamugisha R, Tylleskär T, Tumwine JK, Heggenhougen HK (2012). “Telling my husband I have HIV is too heavy to come out of my mouth”: pregnant women’s disclosure experiences and support needs following antenatal HIV testing in eastern Uganda. J Int AIDS Soc.

[33] Katz DA, Kiarie JN, John-Stewart GC, Richardson BA, John FN, Farquhar C (2009). Male perspectives on incorporating men into antenatal HIV counseling and testing. PLoS One.

[34] Musheke M, Bond V, Merten S (2013). Couple experiences of provider-initiated couple HIV testing in an antenatal clinic in Lusaka, Zambia: lessons for policy and practice. BMC Health Serv Res.

[35] International Fund for Agricultural Development (IFAD). Rural poverty Report 2011. [http://www.ifad.org/rpr2011/media/kit/factsheet_e.pdf]. Access date 05 January 2015.

[36] Ferguson L, Grant AD, Lewis J, Kielmann K, Watson-Jones D, Vusha S (2014). Linking women who test HIV-positive in pregnancy-related services to HIV care and treatment services in Kenya: a mixed methods prospective cohort study. PLoS One.

[37] Fox MP, Shearer K, Maskew M, Meyer-Rath G, Clouse K, Sanne I (2014). Attrition through Multiple Stages of Pre-Treatment and ART HIV Care in South Africa. PLoS One.

[38] Matthews LT, Moore L, Crankshaw TL, Milford C, Mosery FN, Greener R (2014). South Africans with recent pregnancy rarely know partner’s HIV serostatus: implications for serodiscordant couples interventions. BMC Public Health.

